# How Often and How Much? Differences in Dietary Intake by Frequency and Energy Contribution Vary among U.S. Adults in NHANES 2007–2012

**DOI:** 10.3390/nu9010086

**Published:** 2017-01-23

**Authors:** Heather A. Eicher-Miller, Carol J. Boushey

**Affiliations:** 1 Department of Nutrition Science, Purdue University, 700 West State St., West Lafayette, IN 47907, USA; cjboushey@cc.hawaii.edu; 2 Epidemiology Program, University of Hawaii Cancer Center, 701 Ilalo St., Honolulu, HI 96813, USA

**Keywords:** frequently consumed foods, food intake, dietary intake, US adults, energy intake

## Abstract

The objective of this study was to determine the top frequently reported foods or beverages and the top foods or beverages grouped by broad and specific What We Eat In America (WWEIA) categories for adult age groups of 19 to 35 years, 36 to 55 years, and ≥65 years (*n* = 16,399) using data drawn from the cross-sectional, WWEIA, National Health And Nutrition Examination Survey (NHANES) 2007–2012 and to compare intake of broad WWEIA categories ranked by frequency and by energy contribution among these adult age groups. Ranking, unadjusted and weighted frequencies, and the proportion of reported foods or energy out of all reported foods or energy were determined and stratified by age. The Rao–Scott modified chi-square was used to test for significant differences among age groups. Results support dietary quality differences by age; intake of broad WWEIA categories was significantly different among age groups by frequency for alcohol, water, and condiment/sauces. Energy contributions significantly differed among age groups for protein foods, snacks/sweets, and beverages. Frequently reported foods and beverages may be used to inform the creation of search tools used for automatic and user-verified identification of foods and beverages in mobile- or technology-based dietary assessment.

## 1. Introduction

The What We Eat in America/National Health and Nutrition Examination Survey (WWEIA/NHANES) [1] is a rich resource for U.S. dietary information with a history of use for research informing dietary assessment. WWEIA/NHANES data have often been used to ascertain the foods and beverages contributing the greatest proportion of energy or nutrients to the diets of the U.S. population for the purpose of food frequency questionnaire development [1,2] or evaluations of U.S. dietary intake [3,4,5]. Identification of top energy sources is important when the research goal includes developing a list of foods that attempt to capture the most prominent sources of energy and that can be used to compare reports of individuals or groups to determine prominent energy contributions, the essential function of a food frequency questionnaire [5,6,7]. Goals of technology based dietary assessment are different, requiring, most importantly, proper identification of a food regardless of energy or nutrient content and a rapid means of identification.

A list of the most frequently reported foods may improve the speed and accuracy of food and beverage identification for mobile- and technology-based dietary assessment. Dietary search tools for mobile- and technology-based assisted dietary assessment may be improved by integrating frequently consumed food information with food and beverage identification algorithms or in search tools. Just as with traditional dietary assessment, user burden is a crucial consideration [8]. Many applications have been developed for mobile telephones that allow users to record foods in a manner similar to recording food on paper. Emerging image-based methods often require a user to capture an image of food prior to consumption. Once the image is captured, users may then be asked to identify the foods in these images or to confirm an automated identification [9,10]. Consistent among these methods is the availability of a user search mechanism and the desire for the foods to be correctly identified. Given the limited screen space inherent to mobile devices, search tools are a challenge. An optimal search tool requires minimal user input and screen space as well as a tailored list of suggested matches. Scrolling through multiple screens is not acceptable when users may need to identify several different foods with limited time for such tasks. Thus, an abbreviated list of the most frequently reported foods by age group would aid the speed of searches and allow tailoring of other food identification tasks used in dietary assessment or other mobile- and technology-based food identification.

The knowledge of how frequently a specific food or beverage is reported per day in the population may additionally give a more complete picture of how that particular food might influence diet, health, and the patterns of intake. Previous research has not included food rankings by frequency. Rather, foods have traditionally been grouped together for evaluation. Bachman et al. [11] noted this limitation regarding the evaluation of food groups: “…foods were grouped together for ease of presentation that would have been of interest to examine separately [11]”. Identification of daily frequently reported foods could potentially enhance knowledge of “opportunities for shifts in food choices [12]” in the population and allow for “a closer look at current intakes and recommended shifts [12]” as per the 2015 Dietary Guidelines for Americans. 

Differences in diet quality and dietary intake are known to exist among U.S. adults by age [13,14], so desirable lists would be specific to adult sub-groups according to age. Thus, the purpose of this study was to determine the top 25 most frequently reported foods or beverages and the top frequently reported foods or beverages grouped by broad and specific WWEIA food or beverage categories [15] for U.S. adult age groups of 19 to 35 years, 36 to 55 years, and ≥65 years using data drawn from NHANES 2007–2012 and to compare intake of broad WWEIA food or beverage categories ranked by frequency and by energy contribution among these adult age groups. The hypothesis was that food groups would differ in their proportional share of total intake among age groups when ranked by frequency or energy contribution.

## 2. Materials and Methods

### 2.1. Survey Design and Participants

WWEIA/NHANES 2007–2008, 2009–2010, and 2011–2012 were nationally representative cross-sectional surveys continuously conducted by the National Center for Health Statistics (NCHS), a program of the Centers for Disease Control and Prevention (CDC) [1]. The participants of the WWEIA/NHANES were drawn from and are representative of the non-institutionalized and civilian U.S. population. Age, sex, and race-ethnicity were among some of the characteristics used to select participants in the complex multistage, probability sampling framework used in the WWEIA/NHANES. Various subpopulations throughout these 6 years of data were oversampled to allow for the generation of precise and reliable estimates for these groups [16,17]. The NCHS Research Ethics Review Board reviewed and approved NHANES protocol for all NHANES content [18]. The Purdue Committee on the Use of Human Research Subjects deemed the de-identified data and research activities of this study as exempt.

WWEIA/NHANES participants completed an in-depth questionnaire assessing socioeconomic indicators in their homes and diet at the NHANES Mobil Examination Center (MEC) [17]. Age group (19–35 years, 36–55 years, ≥56 years), gender (male or female), survey year (2007–2008, 2009–2010, 2011–2012), poverty–income ratio (0.00–0.99, 1.00–1.99, 2.00–2.99, 3.00–5.00), race/ethnicity (Mexican American and Other Hispanic, Non-Hispanic White, Non-Hispanic Black, and Other-Race including Multi-Race), and body mass index status (normal weight, overweight, and obese [19]) were used to characterize participants included in this study.

Participants were ≥19 years (*n* = 16,399) and completed a 24 h dietary recall during a visit to the MEC. Younger adults aged 19 to 35 years numbered 4703 with 69,366 reported foods that accounted for 1,034,259,606 weighted reports or items consumed in 24 h by the U.S. population age 19 to 35 years. Middle aged adults 36 to 55 years numbered 5476 with 88,385 reported foods that accounted for 1,419,496,272 weighted reports. Older adults aged ≥56 years numbered 6220 with 105,808 reported foods and 1,242,340,208 weighted reports.

### 2.2. Dietary Assessment

The USDA’s Automated Multiple Pass Method (AMPM) [20] 24 h dietary recall was completed during the MEC examination. Approximately 30–45 minutes were necessary to complete the five passes, or series of probes, for retrieving as many foods as possible that were consumed in the past 24 h. The reported dietary information was then linked to the USDA Food and Nutrient Database for Dietary Studies (FNDDS) [21,22,23], a nutrient composition database including information on approximately 7000 foods. The AMPM computerized software system allows for direct coding of the reported foods, data editing and management, and nutrient analysis of the dietary data [20]. The USDA food code assigned to each food listed in the 24 h dietary recall was used to match and sort the reported foods and to assign a WWEIA Food Category [15]. The data of participants without a day-1 dietary assessment or for whom dietary weights were missing were not included in the analysis.

### 2.3. Statistical Analysis

Lists of top reported foods and WWEIA food and beverage categories were stratified and compared by age group—19 to 35 years, 36 to 55 years, and ≥65 years—due to differences in diet expected for different age groups. Unadjusted frequencies were computed by matching and tallying each food code or USDA/WWEIA food or beverage category code, adapting previously used methods [3,4,5,6,7,8,15]:

*n*∑*I* = 1(*Ri*) (1)

where *n* = the sample size, *I* = each participant, and *Ri* = *#* of reports of individual food code or category for the ith individual. Weighted frequencies were computed by determining the weighted sum of each food code or category:

*n*∑*I* = 1(*Riwi*) (2)

where *wi* = sample weight for the ith individual. The weighted proportion of reported foods or the contribution of each food or category reported to the total food or category reports is given as

*n*∑*I* = 1(*Riwi*)/*n*∑*I* = 1(*Tiw*i) (100) (3)

where *Ti* = total *#* of reports of all food codes or food category codes for the ith individual. The weighted percentage of reported energy was determined similarly with substitution of the energy in each reported food or beverage for frequency, and total energy rather than total # of reports was used as the denominator in the previous equation. The Rao–Scott modified chi-square was used to test for significant differences among age groups for characteristics and the weighted proportion of foods and beverages grouped by frequency and by energy contribution and categorized to USDA food and beverage groups. Comparisons for frequently consumed food or beverage categories among age groups were indicated as significantly different when *p* < 0.01/42 or *p* < 0.0002 using a Bonferroni type adjustment for multiple comparisons for food group intake among 14 food groups × 3 age groups to mitigate the probability of finding insignificant results. Survey weights, or the reciprocal of sample inclusion probability, allow for inference to the non-institutionalized U.S. population and were applied to all computations. Further adjustment was made to account for the clustering and stratification inherent to the survey design. Analyses were completed in SAS 9.4 using SAS survey procedures. 

## 3. Results

### 3.1. Characteristics

Age group characteristics varied significantly by gender, poverty–income ratio, race/ethnicity, and body mass index status (Table 1).

Mid adult (36–55 years) and older adult (≥56 years) age groups had slightly less men compared with women; young adults (19–35 years) comprised a higher percentage of those with a low poverty–income ratio compared with mid and older adult age groups. The older adult age group had a higher prevalence of non-Hispanic white and lower prevalence of Mexican American and other Hispanic compared with young and mid adult age groups. Mid and older adult age groups also had a higher prevalence of overweight and obesity compared with young adults. 

### 3.2. Food Group Intake by Frequency and Energy Contribution

Broad intake of WWEIA food or beverage categories was significantly different among age groups by frequency for alcohol, water, and condiments/sauces; the weighted percent of all three groups was greater among younger adult age groups and lessened as age increased (Table 2). Energy contributions significantly differed among age groups for protein foods, snacks/sweets, and beverages. The weighted proportion of total energy accounted for by protein foods and snacks/sweets was greater among older and mid adult age groups compared with younger adults, while beverages showed a reverse pattern. Food categories that differed both by frequency and energy contributions among age groups were mixed dishes, grains, fruit, vegetables, fats/oils, and sugars. All these food categories showed a pattern of higher weighted percentage of reported foods or reported energy among older compared with younger age groups except for mixed dishes which showed a reverse pattern. The weighted proportion of protein foods, mixed dishes, grains, snacks/sweets, and alcohol was higher among all adults by energy contribution compared with reported frequency, but the reverse was true for fruit, vegetables, beverages, water, fats/oils, condiments/sauces, and sugars.

### 3.3. Frequently Consumed Foods

The top 25 most frequently daily reported foods or beverages for each age group are given in Table 3, and the top 25 most frequently consumed specific WWEIA food or beverage categories (out of 150 total categories) are shown in Table 4. Foods are listed by their FNDDS short food descriptions in descending weighted frequency order. Several similarities and differences are notable across age groups and among individual foods and food categories listings. Beverages feature prominently, capturing 11 rankings in the top 25 reported foods or beverages and tap water ranked 1st for both individual food and food category rankings in all age groups. Sweetened drinks ranked higher and more frequently for young compared with mid and older adult age groups in both the individually listed foods and food- or beverage-specific WWEIA category listings. Sugar-free cola-type soft drinks appeared highest ranked among mid aged adults compared with the other age groups. Coffee ranked highly in all age groups but was most frequently consumed among older adult age groups and lower in frequency to mid and young adult groups; unsweetened tea had a similar pattern. Beer showed a reverse age pattern with higher ranking among young followed by the mid adult age group. Milk appeared in older adult lists at a higher frequency and for more varieties of fat percentage compared with mid and younger age groups.

Several foods also ranked among the 25 most frequently consumed foods and beverages; many of these specific foods were common to all age groups, but their rankings varied in the age group lists. Condiments such as tomato catsup, red cooked salsa, mustard, and mayonnaise ranked high among the young and mid adult’s frequently consumed foods, while the older group’s list included only mustard and mayonnaise. Raw fruits and vegetables such as banana, apple, tomatoes, and lettuce were more frequently consumed among older compared with younger adult age groups. White potato French fries and corn or cornmeal tortilla chips were frequently consumed among young adult age groups, only the corn or cornmeal tortilla chips ranked among the mid adult list and neither ranked in the older adult list, a pattern similar to the rankings in the top 25 categories. Meat such as chicken and cold cuts/cured meats were frequently consumed WWEIA food or beverage categories among young adult age groups and showed a declining pattern in frequency as age increased, while nuts/seeds showed a reverse pattern.

## 4. Discussion

Few U.S. dietary reports or studies have included consideration of the contribution of individual food items nor is there previous research featuring intake contributions by both energy and frequency. Significant differences in the proportional share of total intake ranked by frequency or energy contribution were apparent among age groups, meaning that age is associated with the types and importance of certain foods in the overall diet. Consideration of frequency and energy contributions simultaneously results in one of four outcomes for each broad WWEIA food or beverage category considered: both frequency and energy contributions did or did not differ across age groups; otherwise, either frequency differed and energy contributions did not differ across age groups or vice versa. For example, milk/dairy and “other” broad WWEIA category intake were relatively stable by frequency and energy contribution across age groups, while intake of mixed dishes was more frequent and comprised a higher share of energy for younger compared with older adults. The opposite was true for grains, fruit, vegetables, fats/oils, and sugars. These dual differences across age groups are perhaps not surprising. As items are consumed more frequently, they may also make up a greater share of the dietary energy. However, WWEIA categories where only frequency or energy varied across age groups indicate independent differences in the patterns or amounts of intake. For example, beverages accounted for approximately 14% of all foods or beverages reported across age groups, but their share of energy varied from 12% for younger adults, 9% for mid adult, and 7% for older adult groups, indicating that, despite similarities in how often beverages are consumed, caloric value or amount was not similar. Alcohol, water, and condiments/sauces showed a pattern of more frequent use by younger adults compared with mid and older adults, but their share of energy (4%–5%, 0%, and 1%, respectively) was similar across ages. Protein foods and snacks/sweets both accounted for approximately 11%–10% of reported foods or beverages across age groups, but the share of energy comprising these foods increased with age (15%–17% and 13%–17% respectively).

The frequency and energy contribution differences in broad WWEIA category intake across age groups shown in Table 2 support previous findings that older U.S. adults have higher-quality diets compared with younger adults [13,14]. The lists of specific foods and beverages by frequency show individual items that largely contribute to these findings. Several raw fruits and vegetables such as lettuce, apple, tomato, banana, and onion are frequently consumed among the age group lists. In all cases, except for raw onion, these foods ranked higher in older adults compared to young and mid aged adults (Table 3). Soft white rolls and white bread were the most frequently consumed grains among all age groups. Whole wheat bread was the only whole grain making the top 25 frequently reported list and was only listed for older adults. These age-related dietary patterns may be a reflection of differences in group composition by gender, poverty–income ratio, race-ethnicity, body mass, and other unmeasured characteristics. Indeed, the quantified characteristics of the various adult age groups shown in Table 1 indicate significant differences in the representation of gender, poverty–income ratio, race/ethnicity, and body mass index status (*p* ≤ 0.0007), which may impact dietary choices and patterns of intake for the age groups represented in this analysis. Previous research has shown these characteristics to be related to differential energy and nutrient intake from processed foods [24], dietary patterns [25,26], and food group [27,28] as well as by likely differences in taste preferences [29], lifestyles [30,31], and other age-related behaviors [32]. 

Regardless of these likely characteristic and behavior-related associations, dietary differences among adult age groups necessitate tailored and specific dietary assessment search tools to be developed for which the results presented here may be applied. The list of frequently consumed foods can enhance the automated identification of foods and beverages by informing algorithms of the probability of a specific item appearing in an image. Frequency driven lists of beverages, for example, may be used to populate a search specifically for beverages, or beverages that are obscured in image-based identification and most likely to be consumed in a certain type of vessel. The list also informs researchers to the specific foods and beverages and food categories where efforts to identify foods are best placed. The misidentification of top reported items could have a larger effect on overall dietary estimates. For example, researchers may choose to focus development of the most accurate identification methods on those most frequently consumed foods and beverages and those contributing the most energy to total energy intake, or use this information to make decisions along with user preferences or other metadata. 

The broad WWEIA category differences in frequency of intake among age groups may be further specified by the top 25 most frequent and more specific WWEIA food categories reported in Table 4 and the top 25 most frequent individual foods and beverages reported in Table 3. Beverages are prominent in the US diet, accounting for 14.3% of reported items, but may be overlooked because of their lower contributions to energy at 9.6%. The appearance of tap water in the top reported list may not be surprising, and the high ranking of bottled water is similar to previous findings, showing mean intakes inversely associated with age [33]. Milk was a prominent item in all three lists with reduced-fat consistently more frequently consumed among all age groups. Skim milk was more frequently reported among older aged adults compared with young and mid aged adults and older adults also reported greater variety of milk, ranking higher in the frequently consumed list compared with mid and younger age groups (Table 3 and Table 4). Yet, when all milk and dairy was combined to broad WWEIA food categories, differences were not observed (Table 2), perhaps due to the moderating effect of cheese (Table 3) combined with dairy in the broad WWEIA categories shown in Table 2. These diverse rankings by broad WWEIA category, specific WWEIA category, and individual foods show how grouping foods may obscure the importance of certain frequently consumed specific items. Aggregation of foods to broad categories is helpful for a more simplified comparison of intake by age group or other characteristics; however, aggregation may also hide the importance of certain specific items or types of foods that are responsible for much of the difference among age groups. The exclusion or inclusion of certain food or beverage items in a food category has the potential to alter statistically significant comparisons among age groups. Aggregation of foods and beverages to various categorizations may also explain why the results presented here differ from food groups ranked by energy contribution that have been previously published [3,4,5,8].

The list of individual frequently consumed foods reveals dietary items that may be overlooked because of their minimal energy contributions, but may significantly augment the nutrient and non-nutrient profile. For example, condiments and sauces may contain a proportionally large amount of sodium to serving size yet have a very minimal impact on total energy. These foods contribute other components to the diet that may be associated with health or disease. Foods such as tomato catsup, mustard, regular mayonnaise, and salsa ranked among the 25 most frequently consumed items in all age groups (with exception of catsup in older adults). These foods are most likely consumed in small amounts, but due to a high sodium-to-energy ratio [3,21,22,23], the frequent consumption of such condiments may be negatively linked with sodium sensitivity and blood pressure [34]. These foods and the food categories they are represented in showed consistent age-related patterns of use and may be intentionally less frequently consumed among older adults for health reasons.

Diet soda beverages, unsweetened tea, and sugar substitutes are additional frequently consumed items that do not appear as significant contributors to energy. However, the non-nutrient components of these beverages may have some effect on health and diet that may be overlooked when only energy contribution is quantified. The association of the amount and frequency of diet soft drink consumption to health is not well characterized but has been linked with an increased risk of vascular events in a population-based cohort followed over 10 years [35]. The potential for other impacts to health is present given the high frequency of reported consumption. Image-based methods that include automated identification of foods would benefit from information that a cola-type soft drink is more frequently consumed compared with sugar-free, cola-type soft drinks and pepper-type soft drinks and, thus, would more likely be in an image of food. Aggregation of all cola-type soft drinks would obscure the potential discrepancies in energy that these generalizations may impart. Such limits to the accuracy of technology-based dietary assessment may also restrain applicability of technology-based methods to certain studies, e.g., the use of food dyes in soft drinks. Studies focusing on certain dietary components or nutrients, (e.g., use of food dyes in soft drinks, sodium intake, etc.) may be aided by the development of frequently consumed food lists paired with lists of foods and beverages that contribute the most to the intake of that specific dietary component or nutrient. For example, a study designed to monitor sodium intake may reduce error in the calculation of dietary sodium by creating a tailored search for use in mobile- or technology-assisted dietary assessment designed for the study that is focused on the accurate determination of the top foods or beverages contributing to sodium intake or to energy and sodium intake. 

The age-specific lists of frequently consumed foods presented in this paper may be used to improve participant compliance to the often tedious task of dietary assessment using a mobile- or technology-based platform. User burden including time and patience for user-verified food identification may be reduced when search mechanisms integrating frequently consumed foods and beverages are pre-populated with potential matches. Lists may also be further refined to better represent the study participant pool by creating sub-lists by other characteristics. For example, a specific frequently consumed foods list generated for female participants of the Supplemental Nutrition Assistance Program (SNAP) aged 20–30 years may be created to be used in a study focusing on this participant population. The creation of such lists are not tied to NHANES data but may be created from pilot dietary data in specific samples and used independently, or used jointly with NHANES data, to inform mobile- or technology-based dietary assessment. In addition, attention should be paid to the time frame that the lists represent, as dietary intake in a population is constantly changing. 

The NHANES 2007–2012 survey measures and procedures continue to be tested, refined, and updated with scientific advances to ensure currency and quality of this large well-designed and well-executed representative national survey [2]. With regard to the individual frequently consumed food analysis, an inherent limit may be that portion size was not considered; thus, an item usually consumed in tablespoons was equivalent to an item usually consumed in cups. The energy analysis, however, includes the amount due to the consideration of energy, yet the times the food was consumed is not accounted for. Energy was prioritized in this study, but lists of reported foods and beverages prioritized by other dietary components may produce very different results. Other potential limitations of this analysis include possible misreporting, including unreported foods, reporting foods that were not consumed, and under-estimating or over-estimating serving size and amount. Underreported or forgotten foods are most often desserts, sweet baked goods, butter, and alcoholic beverages [36,37]. Improvements in dietary assessment made possible by knowledge of frequently reported foods and beverages go hand-in-hand with improvements in monitoring the foods available in the U.S. food supply. Every year many new items are available for consumption [38], and the results presented here may change over time. Several data sources aiming to monitor these foods are available, but all are limited in scope, accuracy of nutrient information, linkages between data sources, and updates with the most current information [39]. Improvements in the information provided in these database systems will allow for greater precision and accuracy in estimating dietary intake and in improving dietary assessment. 

## 5. Conclusions

Frequency of food group and specific food and beverage intake is an important component of dietary patterns that has not been previously explored. Adult age is associated with certain foods and beverages and their importance in the overall diet. The results of this study may be a starting point to inform future investigation. The list of individual frequently consumed foods may be used to inform consumer education, questionnaire design (such as Food Frequency Questionnaires), and database and search designs for web and mobile applications.

## Figures and Tables

**Table 1:** Characteristics of U.S. adults ≥19 years using NHANES 2007–2012 ^a^.

	All ≥19 Years		19–35 Years		36–55 Years		≥56 Years		
Characteristic	*n*	%	*n*	%	*n*	%	*n*	%	χ^2^ *p*-Value ^b^
**Total**	16,399	100	4703	31	5476	38	6220	31	<0.0001
**Sex**									0.0007 *
Men	8075	49	2342	50	2655	48	3078	49	
Women	8324	51	2361	50	2821	52	3142	51	
**Survey Year**									0.38
2007–2008	5556	34	1482	32	1837	34	2237	36	
2009–2010	5905	36	1685	36	2016	37	2204	35	
2011–2012	4938	30	1536	33	1623	30	1779	29	
**Poverty Income Ratio**									<0.0001 *
0.00–0.99	3441	23	1356	31	1101	22	984	18	
1.00–1.99	4070	27	1179	27	1261	25	1630	29	
2.00–2.99	2208	15	578	13	655	13	975	17	
3.00–5.00	5260	35	1226	28	2008	40	2026	36	
**Race/Ethnicity**									<0.0001 *
Mexican American & Other Hispanic	4242	26	1384	29	1510	28	1348	22	
Non-Hispanic White	7283	44	1772	38	2319	42	3192	51	
Non-Hispanic Black	3568	22	1073	23	1149	21	1346	22	
Other-Race including Multi-Racial	1306	8	474	10	498	9	334	5	
**Body Mass Index Status ^c^**									<0.0001 *
Normal weight & Underweight	4509	28	1772	39	1298	24	1439	24	
Overweight	5381	34	1335	30	1882	35	2164	36	
Obese	6014	38	1418	31	2186	41	2410	40	

^a^ Total numbers do not always add to sample size due to missing values. Percentages do not always add to 100 due to rounding. Estimate represents weighted percent; ^b^ Rao Scott F adjusted χ^2^ *p*-value is shown. Sample weights were appropriately constructed and applied to this analysis as directed by NCHS. Weights were rescaled so that the sum to the weights matched the survey population at the midpoint of the 6 years, 2007–2012. Statistical significance for differences among 19–35, 36–55, and ≥56 year age groups when *p* < 0.001 is indicated by “*”; ^c^ Body Mass Index (BMI) status was classified based on Centers for Disease Control and Prevention BMI values as described in [19].

**Table 2:** Broad WWEIA food category ^a^ intake comparisons by frequency and energy among U.S. adults ≥19 years using NHANES 2007–2012 ^b^.

	Total ≥19 (*n* = 16,399)		19–35 Years (*n* = 4703)		36–55 Years (*n* = 5476)		≥56 Years (*n* = 6220)		χ^2^ *p*-Value	
WWEIA Food Category ^a^	Wtd % ^c^ of Reported Foods (SE)	Wtd % ^c^ of Reported Energy (SE)	Wtd % ^c^ of Reported Foods (SE)	Wtd % ^c^ of Reported Energy (SE)	Wtd % ^c^ of Reported Foods (SE)	Wtd % ^c^ of Reported Energy (SE)	Wtd % ^c^ of Reported Foods (SE)	Wtd % ^c^ of Reported Energy (SE)	Foods ^d^	Energy ^d^
Milk/Dairy ^e^	7.5 (0.1)	6.5 (0.1)	7.6 (0.1)	6.2 (0.2)	7.3 (0.1)	6.4 (0.2)	7.8 (0.1)	7.0 (0.2)	0.02	0.02
Protein ^f^	10.6 (0.1)	16.0 (0.2)	10.6 (0.2)	14.8 (0.3)	10.9 (0.2)	16.6 (0.3)	10.1 (0.1)	16.5 (0.3)	0.0005	<0.0001 *
Mixed Dish ^g^	7.1 (0.1)	20.9 (0.3)	8.8 (0.2)	24.2 (0.4)	7.0 (0.2)	20.5 (0.5)	5.6 (0.1)	17.5 (0.4)	<0.0001 *	<0.0001 *
Grain ^h^	9.4 (0.1)	13.2 (0.2)	9.2 (0.2)	12.2 (0.3)	9.0 (0.1)	12.6 (0.3)	10.1 (0.1)	15.1 (0.2)	<0.0001 *	<0.0001 *
Snack/Sweet ^i^	10.2 (0.1)	15.1 (0.3)	10.1 (0.2)	13.4 (0.3)	10.2 (0.2)	15.4 (0.4)	10.2 (0.2)	16.8 (0.3)	0.92	<0.0001 *
Fruit ^j^	4.6 (0.1)	2.6 (0.1)	3.7 (0.1)	1.9 (0.1)	4.2 (0.2)	2.4 (0.1)	5.8 (0.1)	3.7 (0.1)	<0.0001 *	<0.0001 *
Vegetable ^k^	10.8 (0.1)	5.5 (0.1)	10.0 (0.2)	5.0 (0.1)	10.7 (0.2)	5.6 (0.2)	11.4 (0.2)	6.2 (0.2)	<0.0001 *	<0.0001 *
Beverage ^l^	14.3 (0.1)	9.6 (0.2)	14.3 (0.2)	12.2 (0.3)	14.6 (0.2)	9.3 (0.3)	13.9 (0.1)	6.8 (0.2)	0.01	<0.0001 *
Alcohol ^m^	1.9 (0.1)	4.7 (0.2)	2.1 (0.1)	5.0 (0.3)	2.0 (0.1)	5.1 (0.3)	1.6 (0.1)	3.8 (0.2)	0.0001 *	0.0005
Water ^n^	9.0 (0.2)	0.1 (0.0)	9.8 (0.2)	0.1 (0.0)	8.9 (0.2)	0.1 (0.0)	8.4 (0.2)	0.1 (0.0)	<0.0001 *	0.31
Fat/Oil °	6.2 (0.1)	3.3 (0.1)	5.1 (0.1)	2.6 (0.1)	6.3 (0.1)	3.4 (0.1)	7.0 (0.1)	4.0 (0.1)	<0.0001 *	<0.0001 *
Cond ^p^/Sauce ^q^	4.6 (0.1)	1.0 (0.0)	5.5 (0.2)	0.9 (0.1)	5.0 (0.1)	1.1 (0.1)	3.6 (0.1)	0.8 (0.1)	<0.0001 *	0.01
Sugars ^r^	3.6 (0.1)	1.2 (0.0)	2.7 (0.1)	1.0 (0.1)	3.7 (0.1)	1.3 (0.1)	4.2 (0.1)	1.5 (0.1)	<0.0001 *	<0.0001 *
Other	0.3 (0.0)	0.2 (0.0)	0.3 (0.0)	0.3 (0.1)	0.3 (0.0)	0.2 (0.0)	0.2 (0.0)	0.2 (0.0)	0.04	0.18

^a^ The What We Eat in America broad food categories were applied to categorize all foods and beverages reported in a single day to 14 broad food groups; ^b^ Survey weights and adjustments for the complex survey design were applied to represent the non-institutionalized U.S. population. Total numbers and percentages do not always add up to sample size due to missing values and rounding; ^c^ Wtd % stands for the estimated weighted percent of all reports of foods or beverages or energy from reported foods or beverages reported in a single day that are included in a food group; ^d^ *p*-values were calculated using the Rao–Scott modified chi-square statistic; “*” indicates *p* < 0.01/42 or *p* < 0.0002 using a Bonferroni type adjustment for multiple comparisons for food group intake among 14 food groups × 3 age groups (19–35, 36–55, ≥56 years); ^e^ Milk, flavored milk, dairy drinks and substitutes, cheese and yogurt; ^f^ Meats, poultry, seafood, eggs, cured meats/poultry, and plant-based protein foods; ^g^ Mixed dishes containing meat, poultry seafood; grain-based; Asian; Mexican; pizza; sandwiches, and soups; ^h^ Cooked grains, breads, rolls, tortillas, quick breads and bread products, ready-to-eat cereals, and cooked cereals; ^i^ Savory snacks, crackers, snack/meal bars, sweet bakery products, candy and other desserts; ^j^ Fresh fruits, dried fruits, and fruit salads; ^k^ Vegetables and white potatoes; ^l^ 100% juice, diet beverages, sweetened beverages, coffee and tea; ^m^ Beer, wine, liquor and cocktails; ^n^ Plain water and flavored or enhanced water; ° Butter and animal fats, margarine, cream cheeses, cream, mayonnaise, salad dressings and vegetable oils; ^p^ Condiment; ^q^ Tomato-based, soy-based, mustard, olives, pickled vegetables, pasta sauces, dips, gravies, and other sauces; ^r^ Sugars, honey, jams, syrups, and toppings.

**Table 3:** Top 25 most frequently consumed foods or beverages, unweighted and weighted frequency of reported foods or beverages, percent of reported foods or beverages, and standard error of percent of reported foods or beverages among all reported foods or beverages for U.S. adults aged ≥19 years from 2007–2012 NHANES data.

19–35 Years (*n* = 4703)					36–55 Years (*n* = 5476)				≥56 Years (*n* = 6220)			
Rank	Long Food Code Description	Weighted Frequency ^a,b^	Frequency ^c^	Wtd% ^b,d^ (SD)	Long Food Code Description	Weighted Frequency ^a,b^	Frequency ^c^	Wtd% ^b,d^ (SD)	Long Food Code Description	Weighted Frequency ^a,b^	Frequency ^c^	Wtd% ^b,d^ (SD)
Total		1,034,259,606	69,366			1,419,496,272	88,385			1,242,340,208	105,808	
1	Tap Water	55,604,429	3318	5.4 (0.2)	Tap Water	73,100,233	4150	5.1 (0.2)	Tap Water	69,876,389	5642	5.6 (0.2)
2	Unsweetened Bottled Water	40,258,737	2948	3.9 (0.2)	Unsweetened Bottled Water	44,691,292	3362	3.1 (0.2)	Regular Coffee (from Ground)	40,478,154	3211	3.3 (0.1)
3	Cola-Type Soft Drink	20,627,037	1479	2.0 (0.1)	Regular Coffee (from Ground)	42,706,452	2362	3.0 (0.1)	Unsweetened Bottled Water	28,625,381	3041	2.3 (0.1)
4	Regular Coffee (from Ground)	16,262,044	953	1.6 (0.1)	Granulated or Lump White Sugar	24,982,068	1876	1.8 (0.1)	Raw Tomatoes	21,877,653	1615	1.8 (0.1)
5	Raw Lettuce	16,221,714	1036	1.6 (0.1)	Cola-Type Soft Drink	24,238,426	1639	1.7 (0.1)	Raw Lettuce	19,223,471	1487	1.5 (0.1)
6	Raw Tomatoes	13,731,135	861	1.3 (0.1)	Raw Lettuce	20,962,560	1239	1.5 (0.0)	2% Fat Cow’s Milk	18,277,411	1663	1.5 (0.1)
7	Granulated or Lump White Sugar	12,945,214	995	1.3 (0.1)	Raw Tomatoes	20,741,045	1218	1.5 (0.1)	Granulated or Lump White Sugar	16,234,096	1961	1.3 (0.1)
8	2% Fat Cow’s Milk	12,748,651	835	1.2 (0.1)	Cola-Type, Sugar Free, Soft Drink	17,259,840	789	1.2 (0.1)	Raw Banana	15,782,624	1442	1.3 (0.0)
9	Tomato Catsup	12,504,756	852	1.2 (0.1)	2% Fat Cow’s Milk	15,923,770	1051	1.1 (0.1)	Skim or Nonfat Cow’s Milk	12,679,928	854	1.0 (0.1)
10	Soft White Roll	9,099,759	624	0.9 (0.1)	Soft White Roll	13,733,883	768	1.0 (0.1)	Unsweetened Tea	11,987,297	961	1.0 (0.1)
11	Fruit Flavored, Caffeine Free, Soft Drink	8,993,097	791	0.9 (0.1)	Raw Banana	12,071,824	794	0.9 (0.0)	Cola-Type, Sugar Free, Soft Drink	10,402,021	641	0.8 (0.1)
12	White Potato, From Frozen, French Fries	8,741,934	639	0.8 (0.1)	Tomato Catsup	11,847,152	731	0.8 (0.1)	Soft White Roll	9,915,274	788	0.8 (0.1)
13	Raw Banana	8,676,457	503	0.8 (0.1)	Whole Cow’s Milk	10,830,727	799	0.8 (0.1)	1% Fat Cow’s Milk	9,327,964	697	0.8 (0.0)
14	White Bread	8,246,385	544	0.8 (0.1)	Raw Onions	10,433,323	634	0.7 (0.0)	Whole Cow’s Milk	9,252,622	885	0.7 (0.1)
15	Red, Cooked Salsa	8,236,575	636	0.8 (0.1)	Unsweetened Tea	10,279,210	558	0.7 (0.0)	Cola-Type Soft Drink	9,047,341	870	0.7 (0.1)
16	Whole Cow’s Milk	8,105,183	628	0.8 (0.1)	Skim or Nonfat Cow’s Milk	10,232,014	441	0.7 (0.0)	Raw Apple	8,906,097	771	0.7 (0.1)
17	Beer	7,438,752	518	0.7 (0.1)	Mustard	10,054,274	563	0.7 (0.0)	Raw Onions	8,427,342	656	0.7 (0.0)
18	Cola-Type, Sugar Free, Soft Drink	7,402,709	391	0.7 (0.1)	Raw Apple	9,812,159	621	0.7 (0.1)	Salted Stick Butter	8,306,944	607	0.7 (0.1)
19	Regular Mayonnaise	7,344,705	469	0.7 (0.0)	White Bread	9,737,207	683	0.7 (0.1)	White Bread	8,233,296	833	0.7 (0.0)
20	Corn or Cornmeal Tortilla Chips	7,003,378	450	0.7 (0.0)	Regular Mayonnaise	9,009,240	541	0.6 (0.0)	Decaffeinated Coffee (from Ground)	7,888,243	665	0.6 (0.0)
21	Raw Apple	6,686,214	427	0.6 (0.0)	Beer	8,250,092	602	0.6 (0.0)	Sucralose Based Sweetener	7,400,723	652	0.6 (0.1)
22	Raw Onions	6,586,906	476	0.6 (0.0)	Red, Cooked Salsa	7,854,863	590	0.6 (0.0)	Mustard	7,363,860	581	0.6 (0.0)
23	Mustard	6,575,602	424	0.6 (0.0)	Salted Stick Butter	7,106,006	332	0.5 (0.1)	Regular Mayonnaise	6,765,799	529	0.5 (0.0)
24	Fruit Flavored, Soft Drink	6,516,425	420	0.6 (0.1)	Corn or Cornmeal Tortilla Chips	6,864,705	398	0.5 (0.0)	Peanut Butter	6,509,399	457	0.5 (0.0)
25	Skim or Nonfat Cow’s Milk	5,616,714	278	0.5 (0.1)	Fruit Flavored, Caffeine Free, Soft Drink	6,828,669	602	0.5 (0.0)	Whole Wheat Bread	6,271,446	487	0.5 (0.0)

^a^ The sum of the estimated weighted frequencies for all type foods or beverages reported in one day among adult participants ≥19 years of NHANES 2007–2012; ^b^ Survey weights and adjustments for the complex survey design were applied to represent the non-institutionalized U.S. population; ^c^ The frequency that a food or beverages was reported without dietary weights; ^d^ Derived from the weighted frequency of the individual food or beverage divided by the total weighted frequency of all foods and beverages *(n)* reported in a single day, where *n* = 1,034,259,606 for 19–35 years, *n* = 1,419,496,272 for 36–55 years, and *n* = 1,242,340,208 for ≥56 years. Estimated weighted percent has been abbreviated by “Wtd %”; SD stands for Standard Deviation.

**Table 4:** Top 25 most frequently consumed specific WWEIA food or beverage categories ^a^, unweighted and weighted frequency of reported intake from WWEIA food or beverage categories, percent and standard error of percent of reported WWEIA food or beverage categories among all reported WWEIA categories in U.S. adults aged ≥19 years from 2007–2012 NHANES data.

19–35 Years (*n* = 4703)					36–55 Years (*n* = 5476)				≥56 Years (*n* = 6220)			
Rank	WWEIA Food Category ^a^	Weighted Frequency ^b,c^	Frequency ^d^	Wtd% ^c,e^ (SD)	WWEIA Food Category ^a^	Weighted Frequency ^b,c^	Frequency ^d^	Wtd% ^c,e^ (SD)	WWEIA Food Category ^a^	Weighted Frequency ^b,c^	Frequency ^d^	Wtd% ^c,e^ (SD)
Total		1,034,259,606	69366			1,419,496,272	88,385			1,242,340,208	105,808	
1	Tap water	57,893,320	3461	5.6 (0.2)	Tap water	76,366,453	4350	5.4 (0.2)	Tap water	72,304,700	5862	5.8 (0.2)
2	Soft drinks	45,394,307	3319	4.4 (0.2)	Coffee	61,030,640	3760	4.3 (0.1)	Coffee	65,329,162	5747	5.3 (0.1)
3	Bottled water	40,258,737	2948	3.9 (0.2)	Bottled water	44,691,292	3362	3.1 (0.2)	Yeast breads	47,772,733	4181	3.8 (0.1)
4	Cheese	35,348,152	2205	3.4 (0.1)	Cheese	44,191,063	2509	3.1 (0.1)	Tea	33,523,736	2601	2.7 (0.1)
5	Yeast breads	29,281,422	1904	2.8 (0.1)	Soft drinks	44,146,521	3082	3.1 (0.2)	Cheese	29,359,815	2260	2.4 (0.1)
6	Tomato-based condiments	26,833,874	1923	2.6 (0.1)	Yeast breads	42,343,280	2659	3.0 (0.1)	Bottled water	28,625,381	3041	2.3 (0.1)
7	Coffee	26,277,950	1672	2.5 (0.1)	Tea	32,442,825	1971	2.3 (0.1)	Other vegetables and combinations	25,658,335	2020	2.1 (0.1)
8	Tea	20,015,237	1326	1.9 (0.1)	Sugars and honey	29,495,942	2121	2.1 (0.1)	Lettuce and lettuce salads	25,027,741	1839	2.0 (0.1)
9	Lettuce and lettuce salads	19,402,651	1225	1.9 (0.1)	Diet soft drinks	29,178,618	1377	2.1 (0.1)	Cookies and brownies	23,073,514	1918	1.9 (0.1)
10	Chicken, whole pieces	17,097,727	1316	1.7 (0.1)	Lettuce and lettuce salads	27,086,924	1524	1.9 (0.1)	Tomatoes	22,521,350	1692	1.8 (0.1)
11	Fruit drinks	16,471,153	1376	1.6 (0.1)	Cream and cream substitutes	26,641,935	1587	1.9 (0.1)	Sugars and honey	21,389,371	2359	1.7 (0.1)
12	Other vegetables and combinations	15,824,793	1050	1.5 (0.1)	Tomato-based condiments	26,374,247	1764	1.9 (0.1)	Nuts and seeds	21,299,064	1525	1.7 (0.1)
13	Cold cuts and cured meats	15,597,064	973	1.5 (0.1)	Other vegetables and combinations	25,974,519	1516	1.8 (0.1)	Cream and cream substitutes	20,983,264	1843	1.7 (0.1)
14	French fries and other fried white potatoes	15,535,805	1112	1.5 (0.1)	Chicken, whole pieces	22,641,016	1589	1.6 (0.1)	Milk, reduced fat	19,488,302	1823	1.6 (0.1)
15	Sugars and honey	15,433,108	1137	1.5 (0.1)	Tomatoes	21,501,142	1281	1.5 (0.1)	Diet soft drinks	19,326,599	1359	1.6 (0.1)
16	Cookies and brownies	14,998,884	1044	1.5 (0.1)	Nuts and seeds	20,780,257	1125	1.5 (0.1)	Soft drinks	18,491,167	1910	1.5 (0.1)
17	Rolls and buns	14,726,654	982	1.4 (0.1)	Cold cuts and cured meats	20,759,008	1222	1.5 (0.1)	Sugar substitutes	18,281,444	1650	1.5 (0.1)
18	Tomatoes	14,449,607	907	1.4 (0.1)	Rolls and buns	20,633,962	1184	1.5 (0.1)	Salad dressings and vegetable oils	18,038,142	1291	1.5 (0.1)
19	Salad dressings and vegetable oils	14,065,066	872	1.4 (0.1)	Cookies and brownies	20,282,731	1247	1.4 (0.1)	Margarine	17,363,255	1441	1.4 (0.1)
20	Eggs and omelets	13,461,968	971	1.3 (0.1)	Salad dressings and vegetable oils	19,296,545	978	1.4 (0.1)	Cold cuts and cured meats	17,262,669	1473	1.4 (0.1)
21	Milk, reduced fat	13,450,309	892	1.3 (0.1)	Eggs and omelets	18,422,809	1314	1.3 (0.1)	Eggs and omelets	16,648,111	1616	1.3 (0.0)
22	Pizza	12,798,854	861	1.2 (0.1)	Mustard and other condiments	18,031,726	1114	1.3 (0.1)	Bananas	15,815,748	1443	1.3 (0.0)
23	Beer	12,730,914	844	1.2 (0.1)	Milk, reduced fat	16,788,887	1136	1.2 (0.1)	Rolls and buns	14,822,211	1184	1.2 (0.1)
24	Diet soft drinks	12,568,880	647	1.2 (0.1)	Candy containing chocolate	15,618,112	834	1.1 (0.1)	Chicken, whole pieces	14,620,763	1521	1.2 (0.1)
25	Tortilla, corn, other chips	12,415,106	843	1.2 (0.1)	French fries and other fried white potatoes	15,149,774	971	1.1 (0.1)	Ice cream and frozen dairy desserts	14,536,669	1144	1.2 (0.1)

^a^ The What We Eat in America Food Categories were applied to categorize all foods and beverages to 150 unique categories; ^b^ The sum of the estimated weighted frequencies for all type foods or beverages from WWEIA food categories reported in one day among adult participants ≥19 years of NHANES 2007–2012; ^c^ Survey weights and adjustments for the complex survey design were applied to represent the non-institutionalized U.S. population; ^d^ The frequency that a food or beverages WWEIA category was reported without dietary weights; ^e^ Derived from the weighted frequency of the foods or beverages in a WWEIA category divided by the total weighted frequency of all foods or beverages *(n)* reported in all WWEIA categories in a single day, where *n* = 1,034,259,606 for 19–35 years, *n* = 1,419,496,272 for 36–55 years, and *n* = 1,242,340,208 for ≥56 years. Estimated weighted percent has been abbreviated by “Wtd %”; SD stands for Standard Deviation.
